# Population pharmacokinetics of intravenous colistin sulfate and dosage optimization in critically ill patients

**DOI:** 10.3389/fphar.2022.967412

**Published:** 2022-08-29

**Authors:** Yue-liang Xie, Xin Jin, Shan-shan Yan, Cui-fang Wu, Bi-xiao Xiang, Hui Wang, Wu Liang, Bing-chang Yang, Xue-fei Xiao, Zhi-ling Li, Qi Pei, Xiao-cong Zuo, Yue Peng

**Affiliations:** ^1^ Department of Pharmacy, The Third Xiangya Hospital of Central South University, Changsha, China; ^2^ Department of Pharmacy and Center of Clinical Pharmacology, The Third Xiangya Hospital, Central South University, Changsha, China; ^3^ Department of ICU, The Third Xiangya Hospital of Central South University, Changsha, China; ^4^ College of Pharmacy, Zunyi Medical University, Guizhou, China; ^5^ Changsha VALS Technology Co. Ltd., Changsha, China; ^6^ Sepsis Translational Medicine Key Laboratory of Hunan Province, Central South University, Changsha, China

**Keywords:** colistin sulfate, population pharmacokinetics, urinary recovery, dosing regimens, critically ill patients

## Abstract

**Aims:** To explore the population pharmacokinetics of colistin sulfate and to optimize the dosing strategy for critically ill patients.

**Methods:** The study enrolled critically ill adult patients who received colistin sulfate intravenously for more than 72 h with at least one measurement of plasma concentration. Colistin concentrations in plasma or urine samples were measured by ultraperformance liquid chromatography tandem mass spectrometry (LC-MS/MS). The population pharmacokinetics (PPK) model for colistin sulfate was developed using the Phoenix NLME program. Monte Carlo simulation was conducted to evaluate the probability of target attainment (PTA) for optimizing dosing regimens.

**Results:** A total of 98 plasma concentrations from 20 patients were recorded for PPK modeling. The data were adequately described by a two-compartment model with linear elimination. During modeling, creatinine clearance (CrCL) and alanine aminotransferase (ALT) were identified as covariates of the clearance (CL) and volume of peripheral compartment distribution (V2), respectively. In addition, colistin sulfate was predominantly cleared by the nonrenal pathway with a median urinary recovery of 10.05% with large inter-individual variability. Monte Carlo simulations revealed a greater creatinine clearance associated with a higher risk of sub-therapeutic exposure to colistin sulfate. The target PTA (≥90%) of dosage regimens recommended by the label sheet was achievable only in patients infected by pathogens with MIC ≤0.5 mg/L or with renal impairments.

**Conclusion:** Our study showed that the dose of intravenous colistin sulfate was best adjusted by CrCL and ALT. Importantly, the recommended dosing regimen of 1.0–1.5 million units daily was insufficient for patients with normal renal functions (CrCL ≥80 ml/min) or those infected by pathogens with MIC ≥1.0 mg/L. The dosage of colistin sulfate should be adjusted according to renal function and drug exposure.

## Introduction

In recent years, the global prevalence of nosocomial infections caused by multidrug-resistant Gram-negative bacteria has led to the resurgence of the use of polymyxins (Nang et al., 2021). At present, there are three kinds of polymyxins used clinically, namely polymyxin B, colistin sulfate and colistin methanesulfonate sodium (CMS) (You-ning et al., 2021). Over the past decade, numerous studies have verified significant individual variability in plasma concentrations of polymyxins, especially among critically ill adult patients (Nation et al., 2016; Kubin et al., 2018; Wang et al., 2020; Li et al., 2021b). Because the pathophysiological changes of critically ill patients resulted in extreme inter- and intra-individual pharmacokinetic (PK) variability of most drugs (Abdul-Aziz et al., 2020), it was recommended that pharmacokinetics/pharmacodynamics (PK/PD) principles of antibiotics should be followed for optimizing their dosing regimens to maximize efficacy and minimize toxicity and resistance (Evans et al., 2021). Accordingly, the study on the pharmacokinetics of polymyxins is crucial for optimizing therapy regimens in critically ill patients.

Colistin sulfate, the active form of colistin, has been widely used in China since 2019 (Yu et al., 2022; You-ning et al., 2021). To date, there are limited data on pharmacokinetic properties of colistin sulfate in humans. In animal studies, the pharmacokinetic properties of colistin sulfate are closer to those of polymyxin B than those of CMS. CMS is the form of inactive prodrug which needs to be converted into the active moiety (colistin) to exert its bactericidal effect (Al-Khayyat and Aronson, 1973; Sivanesan et al., 2016). The current understanding of colistin sulfate pharmacokinetics in patients is based on one study (Yu et al., 2022), in which the PK parameters of colistin sulfate were reported to be similar to those of polymyxin B. In that study, plasma samples were used to describe the pharmacokinetic characteristics of colistin sulfate and creatinine clearance (CrCL) was identified as a covariate for the clearance of colistin sulfate. Because urine samples were not collected, it remained unclear whether colistin sulfate could be eliminated by kidney and whether a dosage adjustment was required based on renal function. Therefore, it is worth further investigating the pharmacokinetic characteristics of colistin sulfate in patients and how dosing recommendation might vary across the range of renal functions.

In view of the above, there is an urgent need to investigate the PK/PD of colistin sulfate in critically ill patients and to optimize its clinical application. In this study, plasma and urine samples were collected to build a population PK model as well as to investigate the urinary excretion of colistin sulfate following intravenous administration. The objectives of the current study were to identify patient factors influencing PK, to explore the correlation of PK/PD index with therapeutic efficacy and then to propose dosage regimens of colistin sulfate using Monte Carlo simulation. The findings of this study will provide important new information on the renal handling of colistin sulfate and dosage regimens for clinical use.

## Materials and methods

### Study design and patient population

This study was conducted at the Third Xiangya Hospital of Central South University between May 2021 and April 2022. The design of this research fully conformed to the principles of the Helsinki Accords, and the study protocol was approved by the Research Ethics Committee of the Third Xiangya Hospital of Central South University (No: 2021-S396). Written informed consent was obtained from all patients or their legal representatives. Adult patients (aged ≥18 years) who had received intravenous colistin sulfate (Colistin, Asia Pioneer Pharmaceutical Co., Ltd., Shanghai, China) for ≥72 h with at least one colistin concentration measurement were eligible for inclusion.

Colistin sulfate treatment was at the discretion of their medical teams. In clinical practice, colistin sulfate was administered at 1.0–1.5 million IU (10,000 IU = 0.44 mg) per day divided into 2 or three doses of infusions over 60–120 min. Colistin sulfate was routinely subjected to therapeutic drug monitoring (TDM) in our hospital. Blood samples (2 ml each) for colistin sulfate TDM samples were acquired immediately before starting and upon completing the infusion after at least 3 days of therapy. Other blood samples were collected randomly from residual blood of blood gas analysis or routine blood tests. In addition, urine samples from 6 patients with catheters in the intensive care unit were collected and recorded across a dosage interval (0–12 h after dosing) after 72 h of therapy. All samples were centrifuged for 10 min (4,000 ×g), and the supernatant was immediately collected and stored at −80°C pending analysis. The therapeutic regimen of colistin sulfate, including dosage, dosing interval and infusion duration and sampling time, was recorded in detail for each patient.

The demographic information and clinical variables were retrieved from the computerized medical records, including age, sex, weight, infection diagnosis, comorbidities, acute physiology and chronic health evaluation (APCHE II), liver function indices (such as alanine aminotransferase (ALT), aspartate aminotransferase (AST), total bilirubin (TBIL), direct bilirubin (DBIL), total bile acid (TBA), total protein (TP) and albumin (ALB)), renal function indices (such as uric acid (UA), blood urea nitrogen (BUN) and creatinine clearance (CrCL) estimated by the Cockcroft-Gault equation (Cockcroft and Gault, 1976)), routine blood tests (e.g., white blood cell (WBC), neutrophilic granulocyte percentage (NEUT), hemoglobin (HGB), red blood cell (RBC), hematocrit (HCT), platelet count (PLT)), coagulation indices (such as prothrombin time (PT), thrombin time (TT), activated partial thromboplastin time (APTT), prothrombin activity (PA), fibrinogen (FIB)), C-reactive protein (CRP), procalcitonin (PCT), lactic acid (LAC) value and 24-h urine volume.

### Quantification of colistin sulfate concentrations

Colistin A and colistin B in plasma and urinary samples were quantified using a validated ultraperformance liquid chromatography tandem mass spectrometry (LC-MS/MS) assay. Briefly, samples were pretreated with acetonitrile, and 1 μL supernatant was injected into the LC-MS/MS instrument for analysis. A Waters XBridge BEH HILIC (4.6 × 150 mm, 2.5 μm) column was used. The mobile phase, consisting of eluents A (0.1% formic acid in water, vol/vol) and B (methanol), was delivered at a flow of 0.5 ml/min. Analysis of independently prepared quality control samples indicated good reproducibility (coefficients of variation ≤12.57%), and accuracy ranged from 88.52% to 109.43%. The limit of quantification was 0.034 mg/L for colistin A and 0.059 mg/L for colistin B. Since colistin A and B had similar structures, molecular weights and pharmacokinetic characteristics, the concentrations of colistin A and B were added together and referred to as colistin (Li et al., 2003; Couet et al., 2011).

### Population pharmacokinetic modeling

Population PK analysis was performed using a non-linear mixed-effect modeling with the Phoenix^®^ NLME software (version 8.3.4, Certara L.P. United States). The first-order conditional estimation-extended least-squares (FOCE-ELS) method was used to develop the PPK model. One- and two-compartment models with linear elimination were compared based on Akaike information criteria (AIC) and Bayesian information criterion (BIC). The inter-individual variability of PK parameters was assumed to follow a log-normal distribution and described using an exponential model, and residual variability was described by an additive, proportional, or combined error model. The coefficient of variation (CV%), goodness-of-fit plots, and the likelihood ratio test (-2 loglikelihood; -2LL) were used to evaluate the model.

Then, a stepwise covariate modeling (SCM) method was used to explore candidate covariates on PK parameters, including age, sex, weight, ALT, AST, TBIL, DBIL, TBA, TP, ALB, UA, BUN, CrCL, WBC, NEUT, HGB, RBC, HCT, PLT, PT, TT, APTT, PA, FIB, CRP, PCT, LAC, and 24-h urine volume, where continuous covariates (e.g., age) were modeled by using the power function and categorical covariates (e.g., sex) were described by an exponential function. By comparing with the base model, a drop in objective function value (OFV) > 3.84 (*p* < 0.05, df = 1) during forward selection steps and an increase of OFV >6.63 (*p* < 0.01, df = 1) in backward elimination steps were the inclusion criteria for covariates.

Finally, the reliability of the final model was evaluated by goodness-of-fit plots. Additionally, the performance of the final model was assessed by a prediction-corrected visual predictive check (pcVPC) of 1,000 replicates using Monte Carlo simulations, and a bootstrap analysis with 1,000 samples was conducted to evaluate the model stability.

The renal clearance (CL_R_) of colistin was calculated as the ratio of the amount recovered in urine to the area under the plasma concentration-time curve (AUC) during the same period (Sandri et al., 2013). The percentage of colistin sulfate excreted in urine was defined as the amount of colistin recovered in urine divided by the dose administered. The fraction of renal clearance was estimated by CL_R_ divided by total body clearance (CL) (Li et al., 2003).

### Monte carlo simulation

Based on the final population model, Monte Carlo simulations with 1,000 replicates were performed for 12 clinically relevant dosing regimens at different MICs ranging from 0.5 to 2 mg/L with various renal functions (CrCL: 10, 50, 80, 120 ml/min). The 12 fixed dosing regimens were a loading dose of 1 million units, 1.5 million units or no loading dose, followed by 500, 000 units q12h, 500, 000 units q8h, 750, 000 units q12h or 1.0 million units q12h. The infusion time for a dose was set as 2 h.

The AUC for each dosing regimen was calculated at 24 or 72 h after therapy to evaluate the probability of target attainment (PTA) of *f*AUC/MIC ≥20 (Dudhani et al., 2010a; Cheah et al., 2015), where *f* is the unbound fraction of colistin defined as 0.49 (Nation et al., 2016). Dosing regimens were considered appropriate if PTA ≥90% was achieved. In addition, the average steady-state plasma colistin concentration (C_ss,avg_) was used to evaluate the probabilities of efficacy and toxicity, and the desired C_ss, avg_ were designed to achieve target attainment rates of >80% for C_ss,avg_ ≥ 2 and <30% for C_ss,avg_ ≥ 4 mg/L (Nation et al., 2016).

### Outcomes

The primary endpoint was the clinical outcome, and the secondary endpoint was the microbiological outcome. Clinical outcomes were defined as valid, if signs and symptoms of infection disappeared or improved on Day 7 after the initiation of colistin therapy; or invalid, defined as any of the following criteria: death related to infection, hemodynamic instability, deteriorated Sequential Organ Failure Assessment (SOFA) score, constant fever >38.5°C, repeat isolation of bacterial phenotypically identical to the index isolate. Microbiological outcomes were defined as clearance, if bacterial culture did not find the original bacteria from the same infection site within the full course of colistin sulfate, or uncleared, defined as repeat isolation of bacteria on or after Day 7 after administration of colistin sulfate. Colistin-induced nephrotoxicity was defined using RIFLE (risk, injury, failure, loss of kidney function, and end-stage renal disease) criteria during treatment (Bellomo et al., 2004).

Receiver operating characteristic (ROC) curves were generated to assess the drug exposure parameters AUC_ss, 0–24h_/MIC, the trough, peak and average concentrations at steady state (C_ss, min_, C_ss, max_ and C_ss, avg_) as predictors of clinical outcome. Then the area under the ROC curve (AUC_ROC_) was calculated to evaluate the correlation between the above parameters and clinical outcome.

## Results

### Patients

A total of 20 patients were enrolled in the study. The demographic data of the patients are summarized in Table 1. Renal functions varied considerably among the patients, and the CrCL ranged from 6.5 to 193.8 ml/min. Two patients received continuous renal replacement therapy (CRRT). Treatment was initiated with a loading dose in four patients, and the most common daily maintenance dose was 1.5 million units divided into 2 or three infusions lasting 1 or 2 h. Carbapenem-resistant *A. baumannii* (50%) and *K. pneumoniae* (30%) were the two most common bacteria isolated from bronchoalveolar lavage fluid, blood, puncture fluid or urine.

**TABLE 1:** Patients’ demographic characteristics.

Characteristics	Value*^a^* (*n* = 20)
Age (year)	60.5 (18–92)
Gender	
Male, %	12 (60%)
Female, %	8 (40%)
Total body weight (kg)	55 (45–65)
Daily dose*^b^*	
2.0 MU, %	4 (20%)
1.5 MU, %	17 (85%)
1.0 MU, %	2 (10%)
Estimated CrCL (ml/min)	48.8 (6.5–193.8)
ALT (U/L)	37.5 (7–495)
ALB (g/L)	30.8 (22.2–43.3)
APACHE II score	21.5 (8–34)
SOFA score	8 (3–18)
Continuous renal replacement*^c^*	2 (10%)
Underlying disease	
Liver disease	13 (65%)
Surgery	10 (50%)
Chronic kidney disease	8 (40%)
Sepsis	8 (40%)
Solid organ transplantation	4 (20%)
Site of infection	
Pulmonary	16 (80%)
Bloodstream	6 (30%)
Urinary tract	3 (15%)
Abdomen	2 (10%)
Pathogenic bacteria cultures	
*A. baumannii*	10 (50%)
*K. pneumoniae*	6 (30%)
*P. aeruginosa*	2 (10%)
None	4 (20%)
Concomitant antibiotic(s)	
Meropenem	16 (80%)
Ceftazidime/avibactam	3 (15%)
Piperacillin/tazobactam	2 (10%)
Cefoperazone/sulbactam	2 (10%)
Linezolid	2 (10%)
Teicoplanin	2 (10%)

a Values are median (range) or No. (%);

b Dosage regimens were adjusted in three patients;

c The interval between CRRT, and blood samples collection was over 72 h in 2 patients. MU, million units; CrCL, creatinine clearance estimated by the Cockcroft-Gault equation; ALT, alanine aminotransferase; ALB, albumin; APACHE II, acute physiology and chronic health evaluation II; SOFA, sequential organ failure assessment.

### Population pharmacokinetic modeling

Overall, 98 plasma concentration measurements collected after 72 h of therapy were included in the analysis. Among them, 38.8% (*n* = 38) were samples with troughs, 27.6% (*n* = 27) were samples with peaks, and 33.7% (*n* = 33) were random samples. A two-compartment model with linear elimination performed better than a one-compartment model (AIC: 160.2 vs. 174.2, BIC: 183.5 vs. 187.1, respectively). Thus, a two-compartment model with first-order elimination was selected as the base model, and a proportional error model was used to evaluate the residual variability. Next, covariate model building identified CrCL as the effective covariate of central compartment clearance (CL) and ALT as a significant covariate for volume of peripheral compartment distribution (V2). Age, sex, weight, and other clinical variables had no statistically significant relationship with PK parameters. The final PK model equations are shown in Eqs 1–3, where the value of inter-compartmental clearance (CL2) was estimated as 1.71 L/h with no inter-individual variability. The population PK estimate parameters and bootstrap replicates are presented in Table 2, which indicated that the final model had qualified stability.

V(L)=16.1×exp(ηV) (1)

$$\mathrm{V2}\left(L\right)\mathrm{=50}\mathrm{.5 \times}{\left(\mathrm{ALT}/\mathrm{37}\right)}^{0\mathrm{.635}}$$ (2)

CL(L/h)=1.50×(CrCL/57.5)0.353×exp(ηCL) (3)

**TABLE 2:** Parameter estimates and bootstraps results of the final population pharmacokinetic model.

Parameter	Final model				Bootstrap	
	Estimate	CV (%)	95% CI	Shrinkage (%)	Median	95% CI
tvV(L)	16.1	8.70	13.3–18.9	43.6	16.2	12.9–19.0
tvV2(L)	50.5	22.0	28.4–72.6	NA	53.8	24.8–200
tvCL (L/h)	1.50	11.7	1.15–1.84	5.06	1.46	0.69–1.89
tvCL2 (L/h)	1.71	22.0	0.961–2.45	NA	1.78	1.29–3.77
dV2dALT	0.635	16.6	0.426–0.844	NA	0.633	0.146–0.954
dCLdCrCL	0.353	29.8	0.144–0.563	NA	0.373	0.0766–1.12
Interindividual variability						
ω2V	0.0267	66.4	NA	NA	0.0174	0.000–0.113
ω2CL	0.197	29.2	NA	NA	0.175	0.0656–0.803
Residual variability (σ)						
stdev0	0.228	15.4	0.158–0.297	NA	0.221	0.147–0.292

CV%, percent confidence of variation; CI, confidence interval; tvV, typical value of volume of central compartment distribution (V); tvV2, typical value of volume of peripheral compartment distribution (V2); tvCL, typical value of central compartment clearance (CL); tvCL2, typical value of inter-compartmental clearance (CL2); dV2dALT, exponential parameter coefficient of alanine aminotransferase (ALT) to V2; dCLdCrCL, exponential parameter coefficient of creatinine clearance (CrCL) to Cl; ω^2^V, variance of inter-individual variability for V; ω^2^CL, variance of inter-individual variability for CL; stdev0, standard deviation.

**FIGURE 1:**
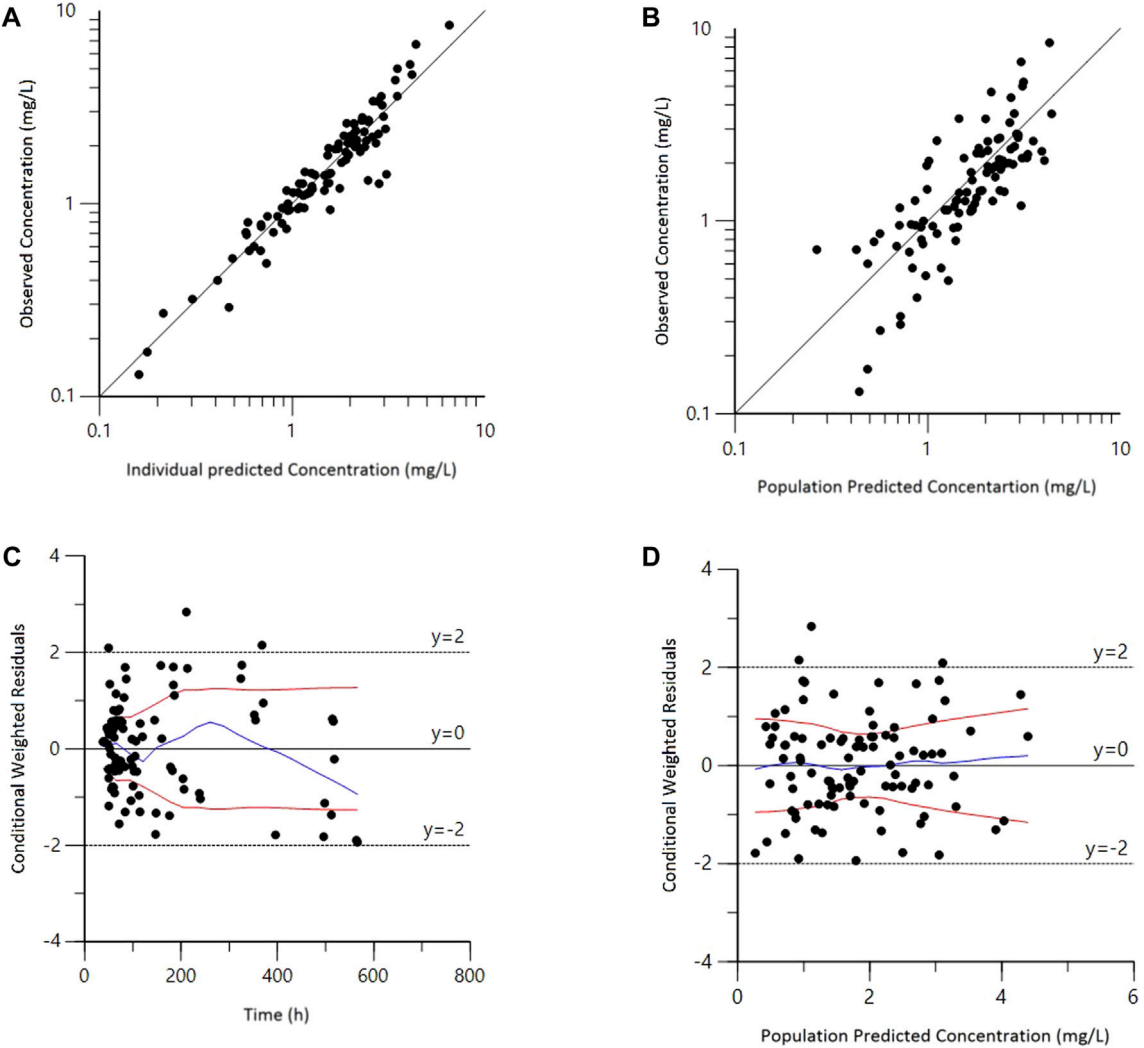
Goodness-of-fit plots for the final population pharmacokinetic model. **(A)** Observed versus population predicted concentrations (DV vs. PRED); **(B)** Observed versus individual predicted concentrations (DV vs. IPRED); **(C)** Conditional weighted residuals versus time (CWRES vs. IVAR); **(D)** Conditional weighted residuals versus population predicted concentrations (CWRES vs. PRED).

**FIGURE 2:**
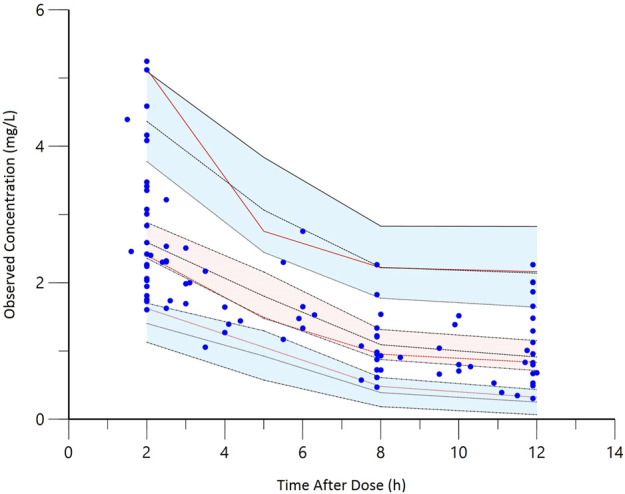
The prediction-corrected visual predictive check (pcVPC) of the final model. The red lines represent the 5th, 50th, and 95th percentiles of the observed concentrations; the shaded areas represent the 80% confidence intervals of the 5th, 50th, and 95th percentiles of the simulated concentrations; the dots represent the observed data.

Goodness-of-fit plots for the final model adequately described the observed data, as shown in Figure 1. The scatterplots of observed concentrations versus PRED and IPRED revealed a perfect correlation. The CWRES versus time or PRED of the final model showed a randomly normal distribution, and most residuals were centered on zero within an acceptable range (−2 to 2). The pcVPC showed that the model reliably predicted the observed data presented in Figure 2, where the most observed plots were within the 90% CIs of the predicted corresponding quantiles. Overall, the results supported the predictive performance of the final model and its use for subsequent dosing simulations.

**FIGURE 3:**
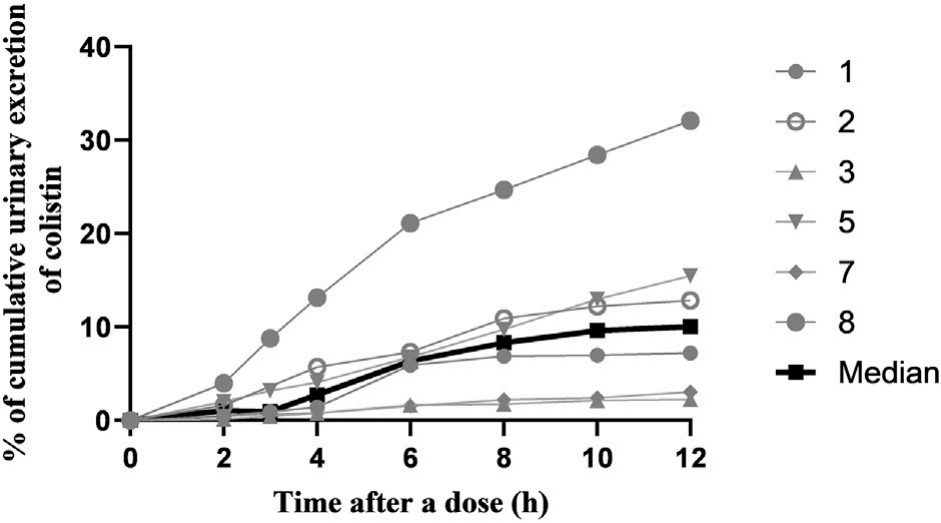
The cumulative urinary excretion percentage of colistin during a dose interval after 72 h of multiple-dose therapy.

A total of 41 urine samples from six patients were used to describe urinary excretion of colistin sulfate. The median percentage of colistin dose that was excreted in urine was 10.05% (range, 2.24%–32.09%), and the median renal clearance (CL_R_) was 0.209 L/h (range, 0.053–0.405 L/h). The CL_R_ of colistin sulfate was a small percentage of CL with large variability (median, 12.37%; range, 2.52%–63.91%) (Figure 3 and Supplementary Table S1).

### Monte carlo simulations

**FIGURE 4:**
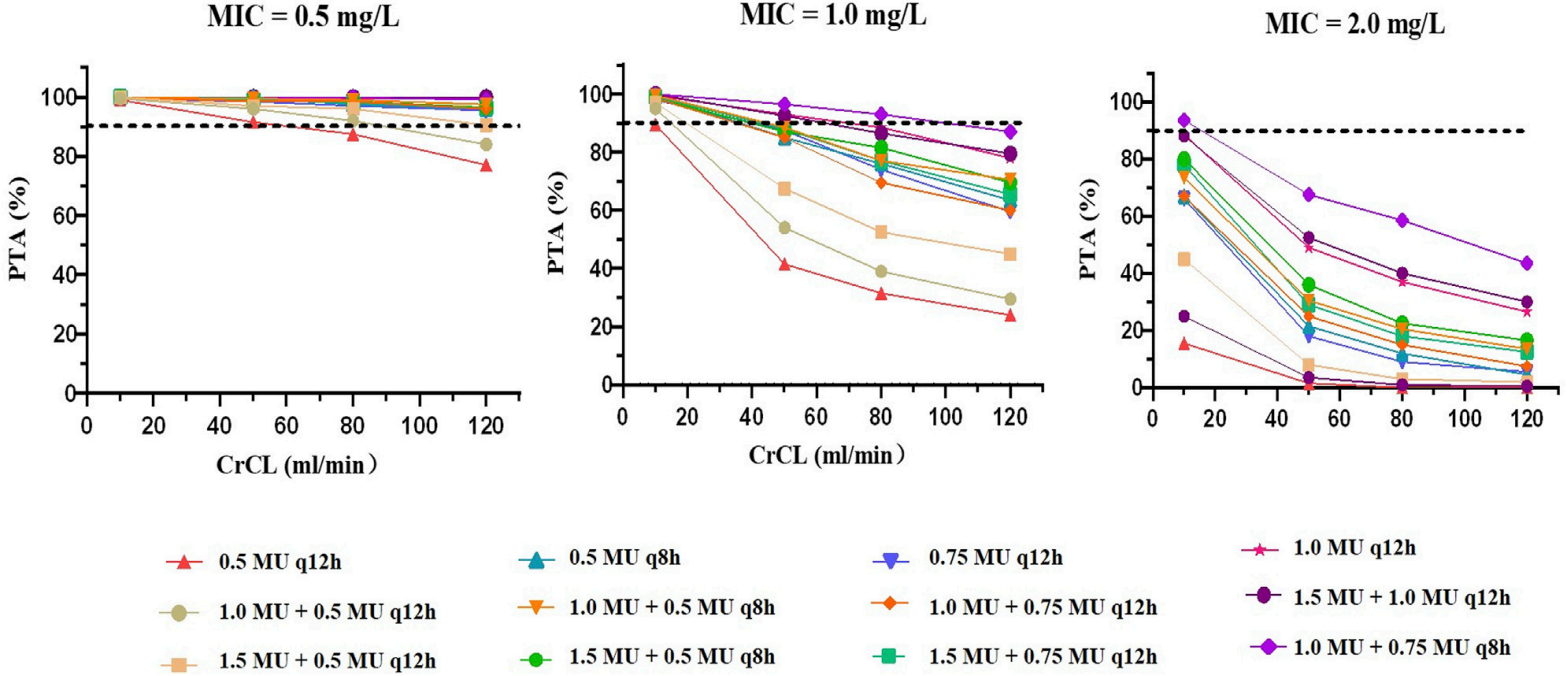
PTAs for dosing regimens performed based on the final PPK model on Day 3. The dotted line represents the target PTA of 90%. A to C show MICs of 0.5 mg/L, 1.0 mg/L and 2.0 mg/L, respectively.

The PTAs of colistin with various dosing regimens when CrCL values ranged from 10 to 120 ml/min, predicted from the Monte Carlo simulations, are presented in Figure 4 and Supplementary Table S2. For an MIC of 0.5 mg/L, the PTAs of almost all simulated regimens were greater than 90% on Day 3, except for the regimen with no loading dose followed by 0.5 MU q12h. When the MIC was less than 1 mg/L, administration of the doses recommended by the label instruction (1.0 MU or 1.5 MU) achieved the PTA over 90% only in patients with CrCL ≤10 ml/min. Yet, when the MIC value was at the current EUCAST susceptibility breakpoint of 2 mg/L, most simulated regimens failed to achieve the target PTA; the only regimen that reached the target PTA was in patients with CrCL ≤10 ml/min treated with 1.0 MU loading dose and maintained on 0.75 MU q8h. Furthermore, we found that a loading dose made it possible to achieve the target PTA on Day 1, which indicated that the loading dose was essential for colistin sulfate treatments.

**FIGURE 5:**
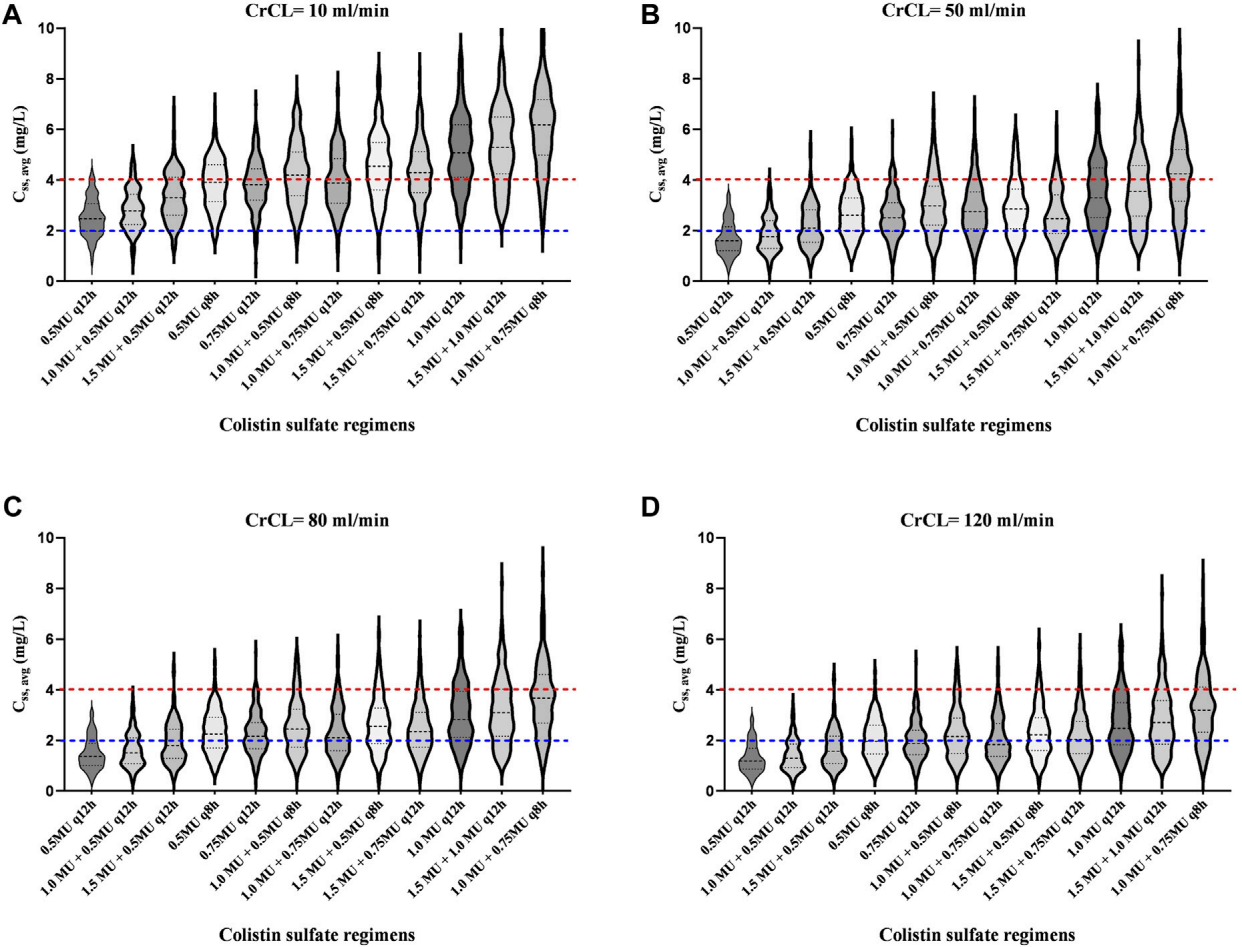
Violin plots of simulation results of average steady-state plasma colistin concentration (C_ss, avg_) for dosing regimens with various CrCL values. The dotted lines represent the interquartile of simulated exposure; the solid lines represent the median of simulated exposure; the red dashed lines represent potential toxicity concentration (4 mg/L); the blue dashed lines represent the target concentration (2 mg/L). A to D show the CrCL values of 10 ml/min, 50 ml/min, 80 ml/min and 120 ml/min, respectively.

In addition, the C_ss, avg_ for various dosing regimens with CrCL ranging from 10 ml/L to 120 ml/min are presented in Figure 5 and Table 3. Based on the simulation results, we conclude that the dose of colistin sulfate should be adjusted according to CrCL. For patients with severe renal insufficiency (≤10 ml/min), a 1.0 MU daily maintenance dose should be selected. For patients with CrCL in 50 ml/min, 80 ml/min and 120 ml/min, the recommended regimen was a loading dose of 1.5 MU followed by daily maintenance doses 1.5, 2.0 or 2.25 MU divided into 2 or three infusions, respectively.

**TABLE 3:** Probability (%) of target Css, avg > 2 mg/L or Css, avg > 4 mg/L for different colistin sulfate regimens.

Dosing regimens	Probability (%) of C_ss, avg_ > 2 mg/L				Probability (%) of C_ss, avg_ > 4 mg/L			
	10 ml/min	50 ml/min	80 ml/min	120 ml/min	10 ml/min	50 ml/min	80 ml/min	120 ml/min
0.5MU q12h	74	29	21	15	4	0	0	0
1.0MU + 0.5MU q12h	85.5	37.5	28	17	7.5	0.5	0	0
1.5MU + 0.5MU q12h	94	53	42	33	28.5	3	1.5	1.5
0.5MU q8h	96.5	75	62.5	49	46	7	2.5	1
0.75MU q12h	96.5	73.5	58	45	41	7	2.5	0.5
1.0MU + 0.5MU q8h	98	77	69.5	56.5	56	16.5	12.5	7.5
1.0MU + 0.75MU q12h	98	70	59	43.5	44.5	12	6	3
1.5MU + 0.5MU q8h	98.5	82	68.5	57	66.5	20	12	7.5
1.5MU + 0.75MU q12h	97.5	81	65.5	52.5	59.5	14.5	7.5	4.5
1.0 MU q12h	99.5	88.5	78	69	76	33	23	13
1.5MU + 1.0 MU q12h	100	87	79.5	71.5	79	39	26.5	16.5
1.0MU + 0.75MU q8h	100	93	87	80.5	87	55.5	36.5	28.5

10, 000 U = 0.44mg; q12h: Every 12 h; q8h: every 8 h; C_ss, avg_: Average steady-state plasma concentration.

### Correlation of drug exposure to outcome

In this study, we evaluated the efficacy of colistin sulfate among 16 patients who received colistin sulfate as definitive therapy; the other four patients who received colistin as empirical therapy could not be assessed in accordance with our evaluation criteria. Overall, clinical outcomes were successful in 12 of 16 cases (75%), and microbiological eradication was observed in 13 of 16 subjects (81.25%) with a causative pathogen MIC range of 0.5–2 mg/L (Supplementary Table S3). None of the patients experienced colistin-induced nephrotoxicity during the treatment. The AUC_ROC_ (0.854, *p* = 0.039) for AUC_ss, 0–24h_/MIC was the largest, indicating that AUC_ss, 0–24h_/MIC had the strongest correlation with efficacy (Supplementary Figure S1). The mean ratio of AUC_ss,0–24h_/MIC was 85.16 h (range 27.49–211.18) and 40.99 h (range 13.83–63.91) in the valid group and invalid group, respectively (*p* = 0.104).

## Discussion

This population PK study has made a significant contribution to understanding the clinical application of colistin sulfate administered intravenously in critically ill patients. To date, there have been limited population pharmacokinetic studies of colistin sulfate in patients (Yu et al., 2022). Our results showed that a two-compartment model with first-order elimination best fitted the PK of colistin sulfate in critically ill patients, which was inconsistent with a previous study in which a one-compartment model with linear-elimination was used to describe the pharmacokinetic characteristics of colistin sulfate (Yu et al., 2022). This could be due to the intensive combined scattered sampling strategy used in the current study (Chen et al., 2022). We collected 6-8 blood samples from six patients across the distribution and elimination phases of colistin sulfate in dosing intervals, which fits to better describe the characteristics of distribution for colistin sulfate in humans (Li et al., 2021a). We also collected urine samples from six patients with catheters among 20 subjects to explore urinary excretion. The urinary recovery of colistin sulfate was low with significant inter-individual variability (median, 10.05%; range, 2.24%–32.09%) in humans, which was higher than those reported in animal studies, in which 0.18 ± 0.14% or 0.13 ± 0.09% of the dose was recovered from urine of Sprague-Dawley rats (Li et al., 2003) or dogs (Al-Khayyat and Aronson, 1973), respectively. Renal clearance of an unchanged drug was a relatively small fraction of total clearance in our study (median, 12.37%), which indicated that non-renal clearance may be the major elimination pathway of colistin sulfate in humans as demonstrated in rats (Li et al., 2003), and was similar to polymyxin B sulfate in patients (Sandri et al., 2013). However, renal excretion of colistin sulfate varied widely among patients. The CL_R_ of patient eight was 63.91%, which was far higher than that of the other patients (Supplementary Table S1). This implied that colistin sulfate was subject to very extensive net reabsorption from tubular urine back into blood, as previously reported in rats (Li et al., 2003) and for polymyxin B in patients (Sandri et al., 2013).

In the present study, the estimated typical value of CL was 1.50 L/h for colistin sulfate, which was lower than the CL of colistin converted from CMS (2.92 L/h) (Couet et al., 2011), and similar to the CL of polymyxin B reported previously (range 1.59–2.86 L/h) (Wang et al., 2021; Chen et al., 2022). CrCL was identified to have a significant effect on the CL of colistin sulfate, which was in agreement with a previous study (Yu et al., 2022) and the disposition characteristic of polymyxin B (Wang et al., 2020; Li et al., 2021b; Yu et al., 2021). Monte Carlo simulations demonstrated that the colistin sulfate exposure showed significant negative correlation with renal function. Accordingly, in patients with renal dysfunction (CrCL ≤50 ml/min), 1.0–1.5 million IU per day divided into 2-3 doses in accordance with the label recommended regimen, would achieve the target therapeutic window. However, in patients with normal renal function (CrCL ≥80 ml/min), a regimen of no less than 2.0 million IU/day would be a better option.

In the current study, we used *f*AUC/MIC ≥20 to evaluate the PTA (Dudhani et al., 2010b), which is widely used to optimize the regimen for polymyxin B (Li et al., 2021b; Yu et al., 2021). The regimen of a 1.0–1.5 million IU daily dose recommended by the current label of colistin sulfate reach PTA ≥90% only for MIC values ≤ 0.5 mg/L, which was in line with a previous study (Yu et al., 2022). High-dose regimens should be considered for the patients infected by organisms with an MIC of ≥1 mg/L. In our limited cases with causative pathogens, all valid patients were infected by organisms with MIC values of 0.5 mg/L, and 50% of invalid patients had a causative pathogen with MIC ≥1 mg/L (Supplementary Table S3). In addition, we confirmed that AUC_ss, 0–24h_/MIC showed a stronger correlation with the clinical efficacy than C_ss, avg_, C_ss, min_ or C_ss, max_ (Supplementary Figure S1).

Moreover, ALT was identified as a significant covariate for volume of peripheral compartment distribution (V2) which explained inter-individual variability; ALT has also been used to describe the pharmacokinetic characteristics of tacrolimus (Lu et al., 2015) and atorvastatin lactone (Dostalek et al., 2012). In our study, because ALT values varied widely from 7 U/L to 495 U/L, the effect of the liver on the disposal of colistin sulfate was considered. Biliary excretion has been verified as one of the pathways for the elimination of polymyxin B (Manchandani et al., 2016) and CMS (Li, 2004) in animal models. We speculated that the liver may also be involved in the disposal of colistin sulfate, which remains to be further explored.

Colistin sulfate and polymyxin B are administered by an active moiety and show similar pharmacokinetic properties. Weight-based dosing strategies for polymyxin B are recommended by international consensus guidelines (Tsuji et al., 2019). However, similar to the study of polymyxin B in Chinese patients (Li et al., 2021b; Wang et al., 2021; Yu et al., 2021), no effect of total body weight on the colistin sulfate PK parameter was observed during modeling in the present study. This may be due to the narrow range of weight values among the included subjects (range, 45–65 kg) and the lack of weight-based doses administered in the clinic, affording low power to detect an effect if present. More research needs to be carried out to explore the relationship between weight and colistin sulfate exposure and whether either weight-based dosing scheme should be used.

Some potential limitations should be considered when interpreting the results of the current study. First, most blood samples were collected from residual blood in the clinic, and therefore, both intensive and sparse sample schedules were used in the study, even though the dosing and sampling time were precisely recorded. Second, the sample size was relatively small, and further evaluation and external validation are needed to confirm the conclusions. Third, we explored urinary colistin sulfate excretion, and large inter-individual variability indicated that urinary excretion should not be ignored. Additional greater sample studies are needed, and TDM is needed for individualized medication. Finally, we evaluated the efficacy and PTA derived from calculated unbound drug fraction rather than measured unbound drug fraction as the previous studies (Wang et al., 2021; Li et al., 2021b; Yu et al., 2022), which may be different from the actual values especially in patients with hypoalbuminemia. Further clinical validation of the regimens based on *f*AUC/MIC simulation is needed, although we evaluated the clinical outcomes and toxicity in limited cases.

## Conclusion

In conclusion, we have presented a reasonable two-compartment population pharmacokinetic model of intravenous colistin sulfate in critically ill patients in China. The study demonstrated that doses of intravenous colistin sulfate are best scaled by CrCL and ALT. Dosage adjustments are recommended according to renal function and drug exposure. Our data indicated that the current dosing recommendation by the label was insufficient for patients with normal renal functions (CrCL ≥80 ml/min) or those infected by pathogens with MIC ≥1.0 mg/L. TDM was recommended to optimize the dosage of colistin sulfate, and further clinical studies on colistin sulfate PK/PD are urgently needed.

## Data Availability

The raw data supporting the conclusion of this article will be made available by the authors, without undue reservation.

## Glossary

TDM: therapeutic drug monitoring
APCHE II: acute physiology and chronic health evaluation
ALT: alanine aminotransferase
AST: aspartate aminotransferase
TBIL: total bilirubin
DBIL: direct bilirubin
TBA: total bile acid
TP: total protein
ALB: albumin
UA: uric acid
BUN: blood urea nitrogen
CrCL: creatinine clearance
WBC: white blood cell
NEUT: neutrophilic granulocyte percentage
HGB: hemoglobin
RBC: red blood cell
HCT: hematocrit
PLT: platelet count
PT: prothrombin time
TT: thrombin time
APTT: activated partial thromboplastin time
PA: prothrombin activity
FIB: fibrinogen
CRP: C-reactive protein
PCT: procalcitonin
LAC: lactic acid
LC-MS/MS: liquid chromatography tandem mass spectrometry
FOCE-ELS: first-order conditional estimation-extended least-squares;
AIC: Akaike information criteria
BIC: Bayesian information criterion
OFV: objective function value
SCM: stepwise covariate modeling
CV%
coefficient of variation
PPK: population pharmacokinetics
DV: observed concentrations
PRED: population prediction
IPRED: individual population prediction
CWRES: conditional weighted residuals
pcVPC: prediction-corrected visual predictive check
CL_R_: renal clearance
AUC: the area under the plasma concentration-time curve
CL: clearance
PTA: probability of target attainment
C_ss,avg_: average steady-state plasma colistin concentration
SOFA: sequential organ failure assessment
RIFLE: risk, injury, failure, loss of kidney function, and end-stage renal disease
ROC: receiver operating characteristic
CRRT: continuous renal replacement therapy
