# Supplementary material for: Population pharmacokinetics of intravenous colistin sulfate and dosage optimization in critically ill patients
Source: Front Pharmacol. 2022 Aug 29;13:967412. doi: 10.3389/fphar.2022.967412 (PMC9465641; doi:10.3389/fphar.2022.967412)
Supplement: Supplementary file 1 [file DataSheet2.PDF]

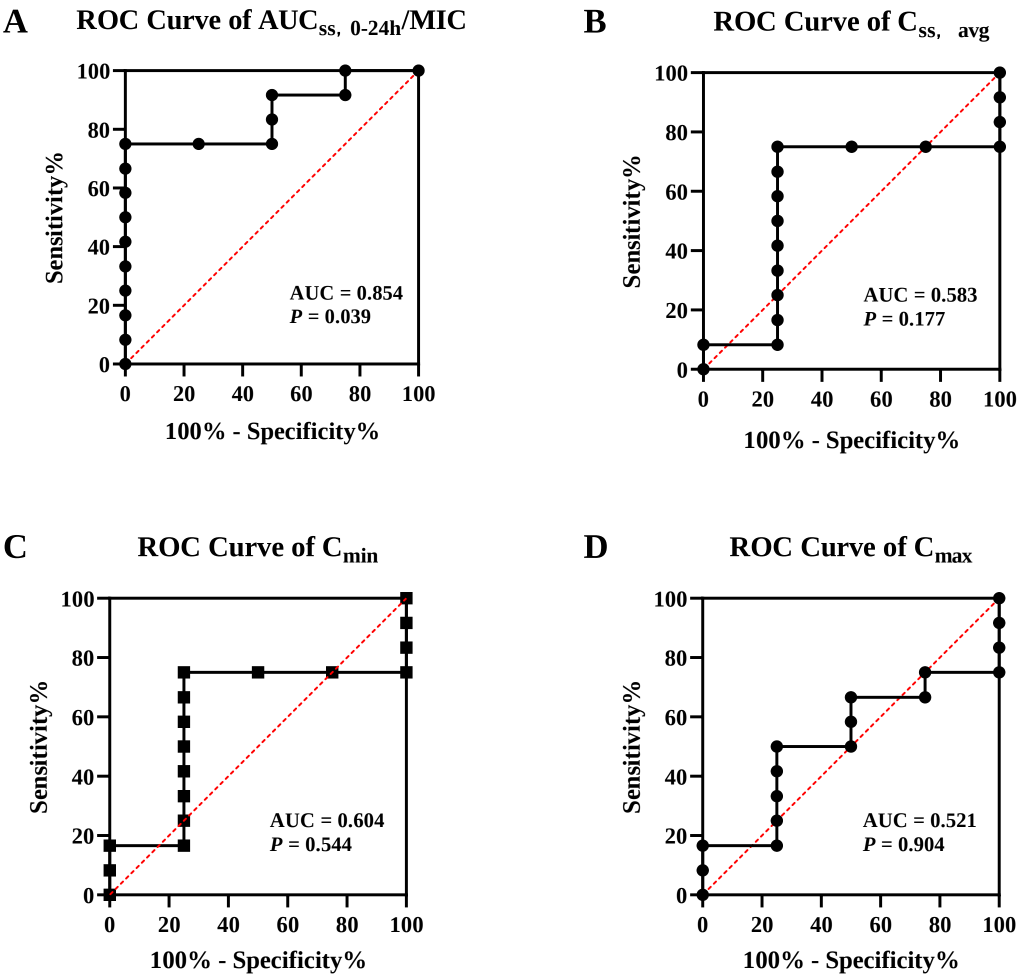

Supplementary Figure 1 The receiver operating characteristic (ROC) curve of colistin sulfate exposure parameters in prediction of clinical efficacy for infections caused by carbapenem-resistant gram-negative bacteria. A to D show ROC curves of $AUC_{ss,0-24h}$/MIC, $C_{ss\ avg}$, C_{min} and C_{max}, respectively.
