# Supplementary material for: Population pharmacokinetics of intravenous colistin sulfate and dosage optimization in critically ill patients
Source: Front Pharmacol. 2022 Aug 29;13:967412. doi: 10.3389/fphar.2022.967412 (PMC9465641; doi:10.3389/fphar.2022.967412)
Supplement: Supplementary file 2 [file DataSheet1.PDF]

Supplementary Table 1 Characteristics of intravenous colistin sulfate excreted in urine

Patient no.	Dosing regimens	Single Dose (mg)	12-hour urine volume (ml)	12-hour urinary excretion of colistin (mg)	Percentage of colistin excreted in urinary (%)	$AUC_{0\text{-}12h}$ (mg·h/L)	CL_R (L/h)	CL (L/h)	CL_R/CL (%)
1	1.0 MU q12h	44.05	1170	3.185	7.23 %	23.93	0.133	1.454	9.15%
2	1.0 MU q12h	44.05	750	5.666	12.86 %	14.00	0.405	2.598	15.58%
3	0.75 MU q12h	33.04	1990	0.740	2.24 %	6.87	0.108	4.269	2.52%
5	0.75 MU q12h	33.04	1620	5.113	15.48 %	17.99	0.284	1.583	17.95%
7	0.75 MU q12h	33.04	540	1.007	3.05 %	19.08	0.053	1.137	4.64%
8	0.75 MU q12h	33.04	1010	10.604	32.09 %	13.83	0.767	1.200	63.91%
	**Median**	33.04	1090	4.149	10.05 %	16.00	0.209	1.519	12.37%

10, 000 U = 0.44mg; q12h: every 12 hours; AUC $_{0\text{-}12h}$: Area under the plasma concentration-time curve over 12 hours; CL: value of central compartment clearance; CL_R: renal clearance.

Supplementary Table 2 PTA for different colistin sulfate regimens based on CrCL and MIC on day 1 and day 3

**Dosing regimens**	**MIC (mg/L)**	**PTA for different CrCL (ml/min) on day 1**					**PTA for different CrCL (ml/min) on day 3**			
		**10**	**50**	**80**	**120**		**10**	**50**	**80**	**120**
0.5MU q12h	0.5	31.5	9.5	5.5	3		99	91.5	87.5	77
	1	0	0	0	0		89.5	41.5	31.5	24
	2	0	0	0	0		15.5	1.5	0	0
1.0 MU + 0.5MU q12h	0.5	98.5	77	66	51		99.5	96	92	84
	1	0	0	0	0		95	54	39	29.5
	2	25	3.5	1	0.5		25	3.5	1	0.5
1.5 MU + 0.5MU q12h	0.5	100	97.5	97	95.5		100	97	96	90.5
	1	60	24	12	6.5		97	67.5	52.5	45
	2	0	0	0	0		45	8	3	2
0.5MU q8h	0.5	93	65	49	38		100	100	97.5	96
	1	0	0	0	0		99.5	85	76	63.5
	2	0	0	0	0		67	21.5	12	4.5
0.75MU q12h	0.5	97	78	65.5	52		99.5	98.5	97	95.5
	1	0	0	0	0		98	87.5	74	59.5
	2	0	0	0	0		66	18	9	5.5
1.0 MU + 0.5MU q8h	0.5	99.5	96.5	93.5	87.5		100	99.5	99	97.5
	1	28	6	3	0.5		99.5	88.5	77	70.5
	2	0	0	0	0		73.5	30.5	20.5	13.5
1.0 MU + 0.75MU q12h	0.5	99.5	89.5	85.5	74		100	98.5	98.5	96
	1	8	0	0	0		98.5	85	69.5	60
	2	0	0	0	0		67	25	15	7.5
1.5 MU + 0.5MU q8h	0.5	100	99	98.5	95.5		100	98.5	98.5	96.5
	1	88	46.5	34.5	19.5		98.5	87	81.5	69.5
	2	0	0	0	0		80	36	22.5	16.5
1.5 MU + 0.75MU q12h	0.5	100	99	97	95.5		100	99	98	96.5
	1	81	32	26.5	18.5		99	88	77	65.5
	2	0	0	0	0		78	29	18	12.5
1.0 MU q12h	0.5	100	96	94	86.5		100	99.5	99.5	99.5
	1	34.5	8	3	2.5		99.5	93	88.5	78
	2	0	0	0	0		88.5	49	37	26.5

1.5 MU + 1.0 MU q12h	0.5	100	100	99.5	96		100	100	100	100
	1	88	46	34.5	25		100	92.5	86.5	79.5
	2	0	0	0	0		88	52.5	40	30
1.0 MU + 0.75MU q8h	0.5	100	97.5	96	94		100	100	100	99
	1	75.5	35.5	19	12.5		100	96.5	93	87
	2	0	0	0	0		93.5	67.5	58.5	43.5

CrCL, creatinine clearance; MIC: minimum inhibitory concentration; PTA, probability of target attainment; gray background: groups that have not reached the target PTA%.

Supplementary Table 3 Pharmacokinetic/pharmacodynamic analysis of colistin sulfate therapy for definitive therapy (n=16)

No.	Site of infection	Pathogen causing infection	MIC (mg/L)	Regimens		CrCL (ml/min)	$AUC_{ss\ 0-24h}$ /MIC ratio	$C_{ss,\ avg}$ (mg/L)	$C_{ss,\ min}$ (mg/L)	$C_{ss,\ max}$ (mg/L)	Concomitant antibiotic(s)	Microbiological outcomes	Clinical outcomes
				Systematic Dosage	Inhaled dosage								
1	Pulmonary	*PA+AB*	0.5	1.0 MU q12h	None	63.93	95.73	1.99	0.94	3.98	MER+LIN	clearance	Valid
3	Pulmonary	*AB*	0.5	0.75 MU q12h	0.25MU q12h	193.84	27.49	0.57	0.13	1.71	MER	clearance	Valid
6	Pulmonary; Bloodstream	*KP*	0.5	1.0 MU q12h	0.25MU q12h	23.79	116.76	2.43	1.95	3.01	PIP/TAZ	clearance	Valid
7	Pulmonary	*KP*	0.5	0.75 MU q12h	0.25MU q12h	10.2	76.34	1.59	0.89	2.49	MER; PIP/TAZ	clearance	Valid
9	Pulmonary	*AB*	0.5	0.75 MU q12h	0.25MU q12h	70.98	97.83	2.04	1.40	3.02	MER	clearance	Valid
10	Pulmonary	*AB*	0.5	0.75 MU q12h	0.25MU q12h	29.3	72.66	1.51	0.87	2.48	MER+LIN	clearance	Valid
11	Bloodstream	*AB*	0.5	1.0 MU q12h	None	26.81	211.18	4.40	3.01	5.84	MER+TEI	clearance	Valid
12	Bloodstream; Urinary tract	*KP*	0.5	0.5 MU q8h	None	9.7	99.20	2.07	1.50	2.67	CEF/AVI	clearance	Valid
13	Pulmonary; Bloodstream	*KP*	0.5	0.5 MU q8h	None	52.49	71.75	1.49	0.96	2.09	CEF/AVI	clearance	Valid
14	Pulmonary; Bloodstream; Abdomen	*KP*	0.5	0.5 MU q8h	None	93.28	35.66	0.74	0.31	1.34	CEF/AVI	clearance	Valid
16	Pulmonary	*AB*	0.5	0.5 MU q8h	0.25MU q12h	45.07	76.04	1.58	1.08	2.24	MER+CEF/SUL	clearance	Valid

18	Pulmonary	*AB*	0.5	0.75 MU q12h	0.25MU q12h	101.48	41.25	0.86	0.28	1.89	MER	clearance	Valid
2	Pulmonary; Bloodstream; Urinary tract	*KP*	0.5	1.0 MU q12h	None	138.05	56.01	1.17	0.43	2.48	MER	clearance	Invalid
8	Pulmonary	*AB*	2	0.75 MU q12h	0.25MU q12h	62.44	13.83	1.15	0.55	2.10	MER	uncleared	Invalid
15	Abdomen	*PA+AB*	1; 2	0.75 MU q8h	0.25MU q12h	41.41	60.42; 30.21	2.52	1.62	3.62	MER+CEF/SUL	uncleared	Invalid
19	Pulmonary	*AB*	0.5	0.5 MU q8h	None	91.55	63.91	1.33	0.83	1.92	MER+TEI	uncleared	Invalid

MIC: minimum inhibitory concentration for colistin; CrCL, creatinine clearance estimated by the Cockcroft-Gault equation; $AUC_{ss, 0\text{-}24h}$, area under the plasma concentration-time curve across 24 hours at steady state; $C_{ss, avg}$: average steady-state plasma concentration. $C_{ss, min}$, minimum concentration at steady state; $C_{ss, max}$, maximum concentration at steady state; MER, meropenem; CEF/SUL, cefoperazone/sulbactam; TEI, teicoplanin; PIP/TAZ, piperacillin/tazobactam; LIN, linezolid; CEF/AVI, ceftazidime/avibactam, *KP: K. pneumoniae; AB: A. baumannii; PA: P. aeruginosa.*
